# Advancing the evidence base for child and adolescent psychopharmacology

**DOI:** 10.1136/bmjment-2025-301634

**Published:** 2025-03-03

**Authors:** Samuele Cortese, Carmen Moreno

**Affiliations:** 1 Developmental EPI (Evidence synthesis, Prediction, Implementation) Lab, Centre for Innovation in Mental Health, School of Psychology, Faculty of Environmental and Life Sciences, University of Southampton, Southampton, UK; 2 Hampshire and Isle of Wight Healthcare Foundation NHS Trust, Southampton, UK; 3 Clinical and Experimental Sciences (CNS and Psychiatry), Faculty of Medicine, University of Southampton, Southampton, UK; 4 Hassenfeld Children's Hospital at NYU Langone, New York University Child Study Center, New York, New York, USA; 5 Department of Precision and Regenerative Medicine and Ionian Area, University of Bari "Aldo Moro", Bari, Italy; 6 Department of Child and Adolescent Psychiatry, Institute of Psychiatry and Mental Health, Hospital General Universitario Gregorio Marañón, IiSGM, CIBERSAM, ISCIII, School of Medicine, Universidad Complutense, Madrid, Spain

**Keywords:** Child & adolescent psychiatry, Depression & mood disorders, Impulse control disorders

## Abstract

This editorial focuses on the seven studies published in the *BMJ Mental Health* topic collection Advances in Clinical Psychopharmacology in Children and Young People. Collectively, these articles provide evidence that informs key steps in the psychopharmacological management of children and adolescents with mental health or neurodevelopmental conditions. Papers in this collection contribute to strengthen evidence-based psychopharmacological practice. We look forward to further developments in the field, supported by adequate research funding.

 Medications are an important component of the multimodal management strategy for many mental health conditions in children and adolescents. Clinical psychopharmacology for children and adolescents is generally considered to have begun in 1937, when Charles Bradley^1^ reported the serendipitous discovery of the beneficial effects of benzedrine, a stimulant, on symptoms that would now be considered under the diagnostic category of attention-deficit/hyperactivity disorder (ADHD). Since then, clinical child and adolescent psychopharmacology has evolved from an art into an evidence-based discipline, driven by the conduct and publication of numerous randomised controlled trials (RCTs) and observational studies. This body of research has significantly contributed to our understanding of the efficacy, effectiveness, and safety/tolerability of medications used to treat mental health conditions in children and young people. However, uncertainties and unmet needs remain in the field.

Seven studies published in the *BMJ Mental Health* topic collection *Advances in Clinical Psychopharmacology in Children and Young People* help strengthen the evidence base for child and adolescent psychopharmacology. Collectively, these articles provide evidence that informs key steps in the psychopharmacological management of children and adolescents with mental health or neurodevelopmental conditions.

First, a study by Lyhmann *et al*^2^ explored a key question that arises after the diagnostic process: *Should the patient be treated with a medication?,* with a specific focus on ADHD comorbidity. The authors applied an advanced quasi-experimental method—instrumental variable analysis—which uses observational data to draw causal inferences. Analysing a sample of more than 8000 Norwegian children and adolescents with ADHD, they examined patients who were ‘on the margin of treatment’— i.e., those whose condition was neither too severe (where most clinicians would prescribe treatment) nor too mild (where most would withhold treatment). This approach allowed them to assess the impact of treatment in the development of subsequent comorbidity in cases where the decision to medicate varied based on provider preference. Among women, ADHD medication nearly eliminated the incidence of reactive disorders over the first 2 years of follow-up. In men, medication significantly reduced the incidence of tic disorders in the first 3 years. However, no other substantial benefits, or harms, of treating those ‘at the margin of treatment’ were observed.

A related key question is: *Should pharmacological or non-pharmacological treatment be used?*. Stringaris and colleagues^3^ addressed this issue focusing specifically on depressive disorders. Current clinical guidelines, such as those from the National Institute for Health and Care Excellence (NICE),^4^ recommend psychotherapy as the first-line treatment for children and adolescents with depression, before considering pharmacotherapy. However, this recommendation is based on indirect comparisons of results from RCTs of psychotherapy and pharmacotherapy. By conducting a meta-analysis of 92 RCTs on psychotherapy and pharmacotherapy for child and adolescent depression, Stringaris *et al* found that psychotherapy control groups often are poorly matched to active intervention arms and hence represent poor and easy-to-beat comparators, as opposed to medication control groups. Therefore, indirect comparisons of results from RCTs of psychotherapy and pharmacotherapy may be problematic. Overall, these somewhat provocative findings challenge the current recommendation that psychotherapy should be the first-line treatment for child and adolescent depression. They also provide a rationale to critically reevaluate and update current clinical guidelines.

When a pharmacological treatment is initiated, an essential aspect is how to measure treatment outcomes. The study by Cohen *et al*^5^ provided highly valuable evidence regarding the ‘minimal important difference’—the smallest change in outcome that is considered meaningful by clinicians—for children with obsessive-compulsive disorder. Using equipercentile linking, the authors determined that a reduction of 5.1 points on the Children’s Yale-Brown Obsessive-Compulsive Scale is required for clinicians to observe minimal improvement.

It is also important to assess whether a pharmacological treatment is prescribed for appropriate indications. In an audit using data from 4151 children and adolescents seen across 59 different National Health Service Trusts in the UK—a country where, unlike other countries, melatonin is available only by prescription rather than as an over-the-counter health supplement of uncertain quality— Paton and colleagues^6^ found that melatonin was generally prescribed for indications supported by an evidence base. Among those prescribed melatonin, 51% had autism spectrum disorder, 42% had ADHD, and 21% had intellectual disability. The remaining patients had diagnoses including anxiety, dissociative, stress-related and somatoform disorders as well as other non-psychotic mental disorders, affective disorders and various forms of insomnia, such as circadian rhythm sleep disorders (including delayed sleep phase syndrome) and other behavioural syndromes. Of concern, among patients who had been prescribed melatonin for over a year, only about two-thirds had their treatment response reviewed in the past year, and in less than half of these cases was the response quantified. Additionally, side effects had been reviewed in fewer than half of those on long-term treatment, and consideration of the need for continued treatment had been documented in less than two-fifths of cases. Another study in the topic collection by Laaksonen *et al*,^7^ a Finnish nationwide retrospective cohort study (1998–2018) involving more than 66 000 patients with paediatric traumatic brain injury (pTBI) and over 60 000 controls with distal extremity fractures, found a significant association between pTBI and post-traumatic ADHD medication use during a 20-year follow-up. This association was particularly prominent after 4 years. The authors suggested that this correlation may be linked to post-TBI neuroinflammation and oxidative stress, which can lead to neurodegeneration. These processes may impact brain development and neurotransmitter function, increasing the risk of neurodevelopmental disorders. Although ADHD medications are not currently licensed for pTBI, the study highlights the need for further research into their efficacy, effectiveness, and tolerability in patients with pTBI.

A crucial aspect of the pharmacological treatment of children and adolescents with mental health conditions is the management of adverse events that may occur during treatment. Underestimating these events poses a potential risk of harm, while overestimating them may lead to inappropriate treatment discontinuation, preventing patients from receiving the benefits of medication. A study in the topic collection further expands our knowledge of the potential adverse effects of ADHD medications. Using VigiBase, the WHO’s pharmacovigilance database, Hamard *et al*^8^ found that, compared with methylphenidate use, amphetamine use was associated with an increased occurrence of reported psychotic symptoms (ROR 1.61; 95% CI 1.26 to 2.06) in patients aged 13–25 years. When the analysis was restricted to ADHD indications, a similar estimate was found (ROR 1.94; 95% CI 1.43 to 2.64). No association was observed for atomoxetine. It is important to note that the study design did not allow the authors to establish causality. However, the findings suggest the importance of monitoring the potential risk of psychosis when prescribing psychostimulants, particularly amphetamines, in clinical practice.

Finally, the topic collection presents epidemiological insights for child and adolescent psychiatry. A cohort study conducted by Jack *et al*^9^ using anonymised electronic health records in primary care from over 5 million individuals (14–28 years) in England (2006–2019) found increased selective serotonin reuptake inhibitors (SSRI) prescribing, depression and anxiety in adolescents (14–18) during autumn. In older age groups (19–28), SSRI prescribing and depression peaked in January, dipped in summer, and rose again in autumn. Possible explanations include teachers advising general practitioner (GPs’) visits at the start of the school year and students delaying care during exams. GPs might also defer recording or prescribing for mental health concerns until they persist. Schools may provide more support during exams, reducing external help-seeking. Additionally, seasonal factors like daylight changes could influence trends. Overall, the study suggests the need of concentrating mental health resources for children and teenagers during autumn, to address increased demand.

While studies in this field continue to advance our understanding of specific aspects related to the pharmacological management of selected neurodevelopmental and mental conditions in children and teenagers, several unmet needs and opportunities remain. These have been recently highlighted in a position paper^10^ by the European College of Neuropsychopharmacology-Child & Adolescent Network, in collaboration with experts by lived experience from national and international associations via a survey involving 644 participants from 13 countries, and representatives from the European Medicines Agency.

Identified key opportunities included:

1. Learning from failed trials, fostering active involvement of individuals with lived experience in different phases of research (from study design to recruitment and dissemination of results), while developing regional research networks to improve recruitment from non-university hospitals and community services.
2. Reducing placebo effect issues—for example, utilising centralised researchers who can help minimise expectation bias.
3. Assessing outcomes beyond core symptoms, including factors such as quality of life.^11^
4. Considering developmental stages in study design, adapting intervention timing to critical developmental windows, including pubertal maturation stages.
5. Comparing pharmacological and non-pharmacological treatments, conducting head-to-head RCTs with placebo and sham arms, followed by summarising evidence through network meta-analyses and umbrella reviews.
6. Exploring innovative trial designs, moving beyond standard RCTs to methodologies such as target-emulated trials^12^ and stepped wedge cluster randomised trials.^13^
7. Advancing precision medicine and stratification approaches, moving away from broad group averages towards individualised treatment prediction.^14^
8. Investigating and implementing digital technologies, including fostering ecological momentary assessment approaches.^15^
9. Focusing on treatment-resistant conditions, addressing conditions that do not respond to initial treatment.
10. Improving the regulatory and legislative framework, encouraging the development of parallel trials for adults and children in conditions with high unmet needs, and designing trials that account for the clinical specificities of paediatric conditions with strong neurobiological underpinnings (eg, targeting irritability across various paediatric conditions).^16^
11. Innovating research approaches, by enhancing international collaboration, sharing and analysing individual participant data from RCTs, promoting the use of standardised nomenclature such as the neuroscience-based nomenclature,^17^ and addressing stigma surrounding pharmacological treatments through coproduced educational and training initiatives led by individuals with lived experience.

Papers in this collection contribute to strengthen evidence-based psychopharmacological practice witj children and adolescents. We look forward to further developments in the field, supported by adequate research funding.
